# Aging causes a reorganization of cortical and spinal control of posture

**DOI:** 10.3389/fnagi.2014.00028

**Published:** 2014-03-03

**Authors:** Selma Papegaaij, Wolfgang Taube, Stéphane Baudry, Egbert Otten, Tibor Hortobágyi

**Affiliations:** ^1^Center for Human Movement Sciences, University of Groningen, University Medical Center GroningenGroningen, Netherlands; ^2^Movement and Sports Science, Department of Medicine, University of FribourgFribourg, Switzerland; ^3^Laboratory of Applied Biology, Faculty for Motor Sciences, Université Libre de BruxellesBrussels, Belgium; ^4^Faculty of Health and Life Sciences, Northumbria UniversityNewcastle Upon Tyne, UK

**Keywords:** postural control, aging, cerebral cortex, fMRI, TMS, neuroplasticity

## Abstract

Classical studies in animal preparations suggest a strong role for spinal control of posture. In humans it is now established that the cerebral cortex contributes to postural control of unperturbed and perturbed standing. The age-related degeneration and accompanying functional changes in the brain, reported so far mainly in conjunction with simple manual motor tasks, may also affect the mechanisms that control complex motor tasks involving posture. This review outlines the age-related structural and functional changes at spinal and cortical levels and provides a mechanistic analysis of how such changes may be linked to the behaviorally manifest postural deficits in old adults. The emerging picture is that the age-related reorganization in motor control during voluntary tasks, characterized by differential modulation of spinal reflexes, greater cortical activation and cortical disinhibition, is also present during postural tasks. We discuss the possibility that this reorganization underlies the increased coactivation and dual task interference reported in elderly. Finally, we propose a model for future studies to unravel the structure-function-behavior relations in postural control and aging.

## Introduction

**Figure 1:**
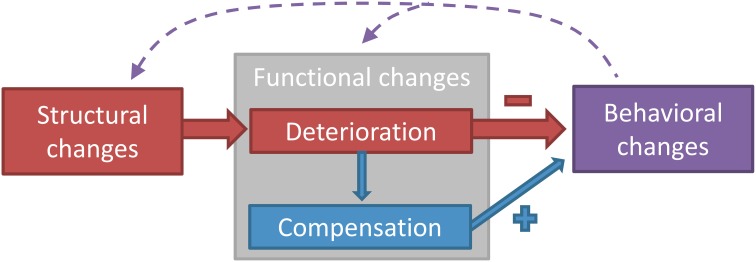
**A classification model of the different domains of age-related changes in the neuromotor system controlling postural and manual tasks**. Three domains can be distinguished: structural changes, functional changes, and behavioral changes. Structural changes refer to the degeneration of brain or nerve structures with aging, whereas functional changes refer to the age-related modification in how these structures operate in the act of motor control. Behavioral changes denote the changes in performance on the motor task, which can be both a postural or a manual task. Functional changes can be divided into deterioration (as a direct result of the structural changes) and compensation (changes in function to counteract the deterioration). Structural degeneration causes functional deterioration (Rivner et al., 2001), which triggers the need for functional compensation (Mattay et al., 2002). Functional deterioration has a negative impact on performance (Nardone et al., 1995), whereas functional compensation has a positive impact (Mattay et al., 2002). The dashed arrows acknowledge the influence that acute or chronic behavioral changes, i.e., intervention or differences in lifestyle, have on structure and function of the neuromotor system (Taube et al., 2007; Rovio et al., 2010; McGregor et al., 2011). The model can be used in future studies to systematically examine the structure-function-behavior link in the aging neuromotor system and could also be applied to other fields of research.

The aging neuromotor system endures structural and functional changes that induce adjustments in motor output. Figure 1 depicts the different domains of age-related changes in the neuromotor system controlling postural and manual tasks. Structural changes refer to a quantitative and qualitative degeneration of gray and white matter and peripheral nerves, whereas functional changes refer to modifications in how these structures operate during a motor task. Functional changes can either be negative (a functional deterioration) or positive (a compensation for the functional deterioration) (Bernard and Seidler, 2012). The extent to which compensation manages to restore function will eventually determine the magnitude of behavioral changes, measured as the change in performance in a motor task.

The preponderance of studies examining the cascade of structure-function-behavior in the aging neuromotor system has used simple manual tasks as a model (Ward and Frackowiak, 2003; Heuninckx et al., 2008; Fling and Seidler, 2012; Fujiyama et al., 2012; Heise et al., 2013). Although tasks such as index finger abduction are relevant to study how aging affects motor control under specific experimental conditions, a simple extension of those findings to complex motor tasks such as those associated with activities of daily living that involve the trunk and the lower extremities would be ecologically invalid. Therefore, the purpose of the present paper is to review the age-related structural and functional changes of the neuromotor system on spinal and cortical level and examine whether these changes are linked to the declines in postural control. Subcortical structures also play a role in postural control (Horak and Diener, 1994; Ouchi et al., 1999; Visser and Bloem, 2005), and age-related changes in subcortical white matter integrity, gray matter volume, and striatal dopaminergic denervation have been shown to affect postural performance. (Cham et al., 2007; Rosano et al., 2007; Murray et al., 2010). Nonetheless, due to a paucity of data on age-related changes in subcortical control of posture, we limited the present review to spinal and cortical mechanisms of postural control.

The concept of postural control can be viewed in a broader perspective, however, for this review we define postural control as the control of upright standing in various conditions. Postural control can be distinguished according to feedback and feedforward control. Feedback control is an ongoing loop of acquisition and integration of sensory information that becomes online updated and corrects posture accordingly during unperturbed and perturbed standing. Feedforward control is an anticipation of potential disturbances and it occurs in anticipatory postural adjustments before a voluntary movement (Woollacott et al., 1984), in context-dependent adaptations of postural responses to perturbations (Horak et al., 1989), and in normal standing (Morasso and Schieppati, 1999; Loram et al., 2005). As during postural control feedforward and feedback mechanisms are working simultaneously and cooperatively, it is often impossible to divide the literature according to this distinction. However, where possible, contributions of the two control mechanisms will be mentioned.

First we review evidence suggesting a role for the frontal, parietal, and motor cortices in the control of standing in healthy young and old adults. Next, we provide an analytical review on how structural and functional changes in the aging neuromotor system mediate behavioral changes in general. The main analysis then focuses on the age-related changes in the structure-function relationship with respect to posture. The last section summarizes evidence for the plasticity in the mechanisms that mediate adaptations to balance training with the goal to slow if not halt the age-related decline in postural control.

## Cortical control of posture

Although classical experiments suggested that the neural control of posture in mammals and primates predominantly relied on spinal reflexes (e.g., Sherrington, 1910), recent studies suggest the involvement and importance of cortical areas in the control of posture during upright standing (for reviews see Jacobs and Horak, 2007; Taube et al., 2008). Behavioral observations in healthy adults, data from patients with cortical lesions, and studies using electro–physiological methods and brain imaging all suggest that postural control involves cortical structures, demonstrating how age-related structural and functional changes in the cortex affect postural control.

Behavioral experiments demonstrate that as soon as subjects receive information about the magnitude of an oncoming postural disturbance, healthy subjects scale and adapt their postural responses according to this information. When subjects receive false cues about a postural disturbance and subsequently over- or underestimate the actual size of the disturbance, the short latency EMG and center of pressure responses are also, respectively, overscaled and undersized (Horak et al., 1989). It may be assumed that these complex feedforward adaptations of postural responses are dependent on the cerebral cortex. In addition, when cognitive demands increase so that the cortical network is charged with the processing of extra workload like in dual- or multitasks, postural performance, quantified by center of pressure measures, decreases (see section Age-Related Reorganization of Postural Control). Impairments in executive functions such as attention, mental calculation, orientation, and memory interfere with balance control, providing additional evidence for the involvement of cortical circuits in postural control (Jacobs and Horak, 2007).

Further evidence to support the importance of cortical structures for the control of posture comes from experiments conducted in patients. Humans and animals with lesions in the sensorimotor cortex and parieto-temporal junction demonstrate abnormal postural control in perturbed and unperturbed standing, like increased body sway, delayed and reduced muscle responses, and absent hopping and placing reactions (Bard, 1933; Brooks, 1933; Diener et al., 1985; Geurts et al., 2005). In addition, balance control, as quantified by the Berg Balance Scale and incidence of falls, correlates with attention deficits in community dwelling cerebral stroke patients (Hyndman and Ashburn, 2003).

Using electroencephalography (EEG), Jacobs et al. (2008) demonstrated that both the activity of the cerebral cortex and the postural reactions adapted in response to cues given prior to rapid horizontal platform perturbations, suggesting that cortical structures are involved in planning postural responses. Similarly, cortical activity was assumed to play a role in preventing falls during voluntary body sway movements as bursts of gamma activity recorded by EEG from frontal and parietal cortical areas were observed to precede the initiation of compensatory postural movements when balance was in danger (Slobounov et al., 2005).

Experiments with transcranial magnetic stimulation (TMS) confirm the involvement of the primary motor cortex when a threat to balance is present. In this respect, Taube et al. (2006) reported increased excitability of the primary motor cortex at the time of the long latency response (LLR) in the soleus muscle after anterior-posterior perturbation. As no effect was seen at the time of the short latency response (SLR) and medium latency response (MLR), it was proposed that the motor cortex becomes involved after approximately 90–100 ms. However, even in unperturbed quiet standing, motor cortical excitability was higher compared to standing while being lightly supported by a board in front of the body (Tokuno et al., 2009). Similarly, short-interval intracortical inhibition (SICI) was comparable in voluntary contractions and upright stance (Soto et al., 2006). Thus, these latter two studies suggest that the primary motor cortex is involved in controlling undisturbed upright stance.

Imaging studies confirm and extend behavioral, EEG, and TMS data concerning the cortical involvement in postural control. A positron emission tomography study revealed increased cortical activity involving the premotor cortex when subjects were asked to imagine themselves standing while they were lying in the scanner (Malouin et al., 2003). Using functional magnetic resonance imaging (fMRI), Zwergal et al. (2012) recorded brain activity in healthy adults age 24–78 when imagining lying, standing, and walking. The basic locomotor and postural networks that were activated consisted of the prefrontal cortex, the basal ganglia, the brainstem, and cerebellar centers. Focusing on the sensory aspects that are important for postural control, Goble et al. (2011) recorded brain activation with fMRI while the dorsal side of the foot was mechanically vibrated at 80 Hz, a stimulus known to excite Ia afferents as part of the proprioception system. There was a positive correlation between the magnitude of activity in parietal, frontal, and insular cortical areas, as well as structures within the basal ganglia in response to vibration, and balance performance during upright stance with eyes closed.

Altogether there is convincing evidence through a variety of approaches that in addition to spinal and subcortical circuits, supraspinal structures like the frontal, parietal, and motor cortices are involved in the control of upright standing. Because age affects the very same structures, it is reasonable to expect an age-related reorganization in the neural control of posture.

## Age-related changes in neural structures involved in postural control

Age induces degeneration of numerous structures in the musculoskeletal, cardiovascular, and nervous system. These structural changes affect functionality of the different systems, forcing a reorganization of the mechanisms that control this functionality so that impairments in motor behavior are minimal (Figure 1). Because the previous section established that postural control involves spinal and cortical structures, this section will outline the structural changes with aging in the nervous system on both levels, in an effort to understand the basis of the reorganization in postural control with aging.

### Age-related structural changes on the peripheral and spinal level

Structural changes in spinal circuits affect their functionality and complicate motor control. We refer to spinal level when sensory and motor neurons and interneurons located in the spinal cord are involved. Morphological studies in aged rodents showed a loss of 38% in myelinated and 46% in unmyelinated fibers of peripheral nerves in the leg, with a decrease in fiber density and myelin thickness and an increase of infolded or outfolded myelin loops (Ceballos et al., 1999; Verdu et al., 2000). Similarly in humans, there is 37 and 38% decline, respectively, in unmyelinated and myelinated fiber density (Jacobs and Love, 1985). The decrease in myelinated fiber numbers and the degeneration of the remaining myelin sheaths are, at least in part, responsible for the 8–18% age-related decrease in nerve conduction velocity (Verdu et al., 2000). Since there is no difference in soleus M-wave latency between young and old adults (5.19 vs. 5.18 ms) despite a delayed H-reflex (29.85 vs. 33.24 ms), it seems that afferent axons and/or synapses are more affected (Scaglioni et al., 2003). Also, there is an age-related reduction in the number of muscle spindles, an important sensory source for postural control (Kararizou et al., 2005). Degeneration of efferent pathways is evident in the tibialis anterior, with a decline of 39% in estimated motor unit number in old (66 years) compared with young (27 years) men along with an even greater decline of 61% in very old (82 years) compared with young men (McNeil et al., 2005). Isometric muscle strength did not decrease beyond age 80, probably because of collateral reinnervation of muscle fibers, increasing the size of the remaining motor units (Stalberg and Fawcett, 1982; Brooks and Faulkner, 1994; McNeil et al., 2005).

### Age-related structural changes on the cortical level

In addition to the morphological alteration of spinal neurons and motor units, cortical neurons also exhibit structural changes that contribute to the evolving dysfunction of the aging neuromotor system. Structural changes on cortical level are related to gray and white matter volume and white matter integrity, parameters most often measured by specific sequences of magnetic resonance imaging. Gray matter consists of neuronal cell bodies, neuropil, specific glial cells, and capillaries. In general, gray matter volume decreases 4–16% with age in a wide range of cortical areas (Bartzokis et al., 2001; Ge et al., 2002; Raz et al., 2004; Salat et al., 2004; McGinnis et al., 2011). The shrinkage occurs in association areas located in the prefrontal and the inferior parietal cortices, as well as unimodal sensory and motor areas (Resnick et al., 2003; Raz et al., 2004; Salat et al., 2004; McGinnis et al., 2011). The physiological changes underlying this cortical thinning are not yet fully understood. However, it is thought that in healthy aging reduced neuronal complexity (neuronal size, synaptic density, presynaptic terminals, etc.) has a greater contribution to cortical thinning than an actual reduction in cell numbers (Morrison and Hof, 1997; McGinnis et al., 2011). The functional relevance of the reduction in gray matter volume for motor performance is evidenced by its correlation with performance declines in mirror drawing (Kennedy and Raz, 2005) and reaching (Sridharan et al., 2012).

White brain matter comprises predominantly glial cells and myelinated axons that connect regions of the cerebrum and the lower brain centers. Aging has a negative effect on white matter quantity, although this volumetric decline starts later and it accelerates faster than the shrinkage of gray matter (Ge et al., 2002). White matter tissue integrity, measured by fractional anisotropy and diffusivity, declines linearly with age at a rate of about 2.5% per decade (Ota et al., 2006; Sullivan and Pfefferbaum, 2006). Brain structure–behavior relationships have been difficult to establish using white matter volumetric measures but have been regularly observed with white matter integrity measures (Sullivan and Pfefferbaum, 2006). The decrease in white matter integrity, primarily in the corpus callosum, that constitutes the largest white matter mass in the healthy human brain (Wahl et al., 2007; Jarbo et al., 2012), has been associated with a slowing of motor performance on interhemispheric transfer tasks like alternating finger tapping (Sullivan et al., 2001). Also central processing speed, examined by choice reaction tasks, correlates with white matter integrity in old adults (Kerchner et al., 2012).

## Age-related functional changes

The structural degeneration causes changes in the function of the affected nerves and neural circuits. These functional changes can either be a deterioration of function as a direct result of the structural changes, or a compensation for this deterioration (Figure 1). When compensating for functional decline, the neuromotor control system reorganizes and changes the contribution of different subsystems. We note that a compensation to improve performance in one task can be maladaptive for a different task. Because of the scarcity of data directly addressing the functional deterioration and compensation that occurs with aging in postural tasks, we review the next best data, and shed light on the reorganization of the neuromotor system during voluntary motor control in old adults. This will be interpolated to postural control in the following section.

### Age-related functional changes on the peripheral and spinal level

The age-related structural changes in the peripheral nervous system cause deterioration in peripheral nerve function, quantified by reduced nerve conduction velocity and response amplitude. Linear regression analysis on data from a group of 3969 healthy subjects between the ages of 20 and 95 years old showed that age explained 3–9% of the variance in nerve conduction velocity and 7–16% of the variance in sensory and motor response amplitude (Rivner et al., 2001). Also the joint position sense in the ankle deteriorates with age with about 3% per year between ages 20 and 80 years, probably as a consequence of the degeneration of muscle spindles (Skinner et al., 1984).

Possibly related to the deterioration in peripheral nerve function, aging seems to cause reorganization in the relative contribution of supraspinal and spinal inputs to the gain regulation of isometric force. To produce a higher isometric plantar flexion force, young adults down regulate their presynaptic inhibition, allowing for an intensified excitatory afferent input. In contrast, old adults show less modulation of presynaptic inhibition, despite their ability to modulate the force (Earles et al., 2001). This suggests that during a voluntary contraction of a leg muscle, old adults rely less on spinal mechanisms (modulation of presynaptic inhibition) and more on supraspinal mechanisms (descending drive) to increase force. It should be noted however, that postsynaptic mechanisms (e.g., recurrent inhibition, Ib inhibition) might have influenced the results.

### Age-related functional changes on the cortical level

Because of the high complexity of brain compared to peripheral nerve function, simple deterioration in brain function is harder to define. However, fMRI and TMS studies have shown many age-related changes in activation and inhibition patterns during voluntary motor control, again demonstrating reorganization.

Right hand movements are mainly controlled by the left hemisphere, because of the cross-over of pyramidal cells to the contralateral side in the medulla oblongata. When healthy young individuals perform a motor task with one hand, brain areas other than the motor network controlling the active hand become also somewhat activated. However, when old adults perform motor tasks with one hand, several brain areas become more strongly activated, including the M1 ipsilateral to the moving hand (Mattay et al., 2002; Ward and Frackowiak, 2003; Langan et al., 2010; McGregor et al., 2011). This increased activation was suggested to be the result of degeneration of the corpus callosum, which generally has a net inhibitory effect on the ipsilateral motor cortex (Sohn et al., 2003). However, Fling and Seidler (2012) found that interhemispheric inhibition is negatively instead of positively correlated with corpus callosum integrity in old adults. Moreover, they found greater interhemispheric facilitation and less interhemispheric inhibition in old compared to young adults. These results suggest a shift from interhemispheric inhibition to excitation in old adults. Most importantly, the decreased interhemispheric inhibition correlated with better motor performance, suggesting that the decreased interhemispheric inhibition served a compensatory purpose for other structural declines. This is in agreement with Mattay et al. (2002), who found longer reaction times in old adults who did not show increased activation in the ipsilateral M1 compared with those who did show increased activation. In contrast, Langan et al. (2010) and McGregor et al. (2011) found that increased ipsilateral M1 activation was associated with longer reaction times in old adults. Hence, increased ipsilateral M1 activation may also be counter-productive and a reflection of non-selective recruitment or de-differentiation. Overall, these studies point to the importance of correlating anatomical and activity related changes in brain structure with motor performance, to be able to understand the functional meaning of the observed cortical changes.

The greater activation in the aging brain during motor tasks is not limited to the ipsilateral M1 and is present in other brain areas, including contralateral M1, prefrontal, and premotor areas (Calautti et al., 2001; Mattay et al., 2002; Ward and Frackowiak, 2003). As most studies agree that increased activation in these regions is associated with better motor task performance in old adults, it seems that the greater activation compensates for structural degradation (Mattay et al., 2002; Wu and Hallett, 2005; Heuninckx et al., 2008) and signifies the allocation of greater neural resources to execute a motor task. Evidence supporting this hypothesis comes from a study showing a positive association between activity of certain brain areas and handgrip force in healthy young adults (Ward et al., 2008). This increase in activation when an increase in force is required was less in old adults in the M1, primary sensory cortex (S1), dorsolateral premotor cortex, and anterior cingulate sulcus, but was higher in the ventrolateral premotor cortex. These results have been replicated in a different cohort (Talelli et al., 2008a), and suggest that at least in this task the ventrolateral premotor cortex is compensating for the lack of activation increase in other areas.

One issue with the above-mentioned fMRI studies is that the interpretation of BOLD (blood-oxygen-level dependence) responses is somewhat limited, as it cannot distinguish between inhibition and excitation (Arthurs and Boniface, 2002). For this purpose, TMS techniques can be used to investigate the excitability of different inhibitory and excitatory circuits within the motor cortical areas. In general, it seems that cortical inhibitory circuits are less active in old compared with young adults. In addition to the already mentioned decrease in interhemispheric inhibition, also shorter silent periods, reduced short-interval intracortical inhibition (SICI) and reduced cortical reciprocal inhibition have been associated with aging (Sale and Semmler, 2005; Hortobágyi et al., 2006; Oliviero et al., 2006; Marneweck et al., 2011; Fujiyama et al., 2012). Table 1 summarizes the age-related changes in cortical inhibitory circuits.

**Table 1:** **Age-related changes in cortical inhibitory circuits**.

**References**	**Muscles**	***N***	**Age**	**Motor tasks**	**IHI**		**cSP**	**cSP/MEP**	**SICI**	**CRI**	**Correlation MP**
					**Type**	**Aging**					
Sale and Semmler (2005)	FDI	y: 10	y: 27 ± 1	(a) 5% MVC			(a) ↓	(a) ↓			Only weak correlations
		o: 10	o: 68 ± 2								
Oliviero et al., 2006	FDI	y: 20	y: 26 ± 4	(r) rest			(a) ↓	(a) =	(r) =		
		o: 22	o: 71 ± 6	(a) 50% MVC							
Talelli et al., 2008b	FDI	30	19–78	(a) 15–20% MVC	IHI10	(a) =					
					IHI40	(a) ↓					
Marneweck et al., 2011	FDI	y: 25	y: 18–29	(r) rest					(r) ↓		No SICI visible: MP ↓
		o: 24	o: 59–88								
McGregor et al., 2011	FDI	y: 15	y: 18–37	(a) 40–50% MVC	iSP	(a) ↓					Trend IHI ↓ MP ↓
		o: 30	o: 60–85								
Smith et al., 2011	FDI	y: 15	y: 20 ± 2	(r) rest					(r) =		
		o: 15	o: 66 ± 4								
Fling and Seidler (2012)	FDI	y: 21	y: 22 ± 3	(a) 20% MVC	iSP	(a)↓/=					IHI ↑ MP ↓
		o: 18	o: 67 ± 5								
Heise et al., 2013	FDI	64	20–88	(r) rest					(r) ↓		SICI rest ↓ MP ↓
				(a) preparation SRT					(a) modulation ↓		SICI modulation ↓ MP ↓
Rogasch et al., 2009	APB	y: 14	y: 21 ± 2	(r) rest					(r) =		
		o: 14	o: 68 ± 6								
Cirillo et al., 2010	APB	y: 12	y: 22 ± 2	(r) rest					(r) =		
		o: 14	o: 67 ± 4								
Hinder et al. (2010)	APB	y: 10	y: 26 ± 3	(a1) tonic	IHI10	(a1) =					
		o: 10	o: 66 ± 4	(a2) ballistic		(a2) =					
Petitjean and Ko (2013)	APB	y: 20	y: 28 ± 7	(a) active	iSP	(a) ↓					
		o: 20	o: 58 ± 7								
Kossev et al., 2002	ECR/FCR	y: 10	y: 29 ± 5	(r) rest					(r) ↑		
		o: 10	o: 56 ± 5								
Hortobágyi et al., 2006	ECR	y: 6	y: 27 ± 4	(r) rest						↓	
		o: 6	o: 73 ± 6								
Fujiyama et al., 2012	ECR	y: 15	y: 18–29	(a1) contralateral ISO			(a1) =				
		o: 15	o: 58–84	(a2) contralateral nonISO			(a2) ↓	(a2) ↓			SP ↓ MP ↓
				(a3) ipsilateral ISO			(a3) =				
				(a4) ipsilateral nonISO			(a4) =				
McGinley et al., 2010	FCR	y: 21	y: 21 ± 1	(r) rest			(a)↑		(r) ↑		
		o: 9	o: 71 ± 2	(a) 15% MVC					(a) =		
Hunter et al. (2008)	BIC	y: 17	y: 26 ± 4	(a) 100% MVC			(a) =				
		o: 7	o: 73 ± 3								
Eisen et al. (1996)	EDC	y: 23	y: 33 ± 7	(a) active			(a) ↓				
		o: 15	o: 67 ± 9								
Lo and Fook-Chong (2004)	AH	30	23–80	(a) 100% MVC	iSP	(a) =	(a) =				
Stevens-Lapsley et al., 2013	VL	y: 20	y: 25 ± 2	(r) rest					(r) =		
		o: 20	o: 58 ± 6								

*Arrows indicate direction of change of cortical inhibition in old compared to young adults. Key: N, number of subjects; IHI, interhemispheric inhibition; cSP & iSP, contralateral & ipsilateral silent period; MEP, motor evoked potential; SICI, short interval intracortical inhibition; CRI, cortical reciprocal inhibition; MP, motor performance; FDI, first dorsal interosseous; APB, abductor pollicis brevis; AH, abductor hallucis; EDC, extensor digitorum communis; BIC, biceps brachii; ECR, extensor carpi radialis; FCR, flexor carpi radialis; y, young; o, old; MVC, maximal voluntary contraction; (a), active; (r), rest*.

The TMS evoked silent period is an interruption of ongoing EMG activity after stimulating the contralateral M1 with TMS, and is believed to reflect the GABA-B mediated cortical inhibition (Werhahn et al., 1999). Although the age-related changes in contralateral silent period reveal some inconsistencies, a general trend is a decrease in inhibition in old compared to young adults with four out of seven studies reporting a shorter and only one study a longer silent period. The inconsistencies can be related to between-study methodological differences, like the muscle investigated and selection of TMS parameters. There is also variation with respect to inhibition measured by the silent period according to task complexity. Execution of a simple wrist movement did not affect silent period duration in young and old adults but the silent period lengthened in young and shortened in old adults when they performed difficult coordination tasks (Fujiyama et al., 2012). Interestingly, within the old group low performers had a shorter silent period than high performers. Thus it might be speculated that the decrease in the silent period with ongoing age might be a compensatory strategy. However, it should be noted that the duration of the silent period correlates with the MEP size (Orth and Rothwell, 2004). To rule out that differences in silent period are caused by differences in MEP size, a ratio between the two should be used. Oliviero et al. (2006) found that the shortening of the silent period with aging disappears when the silent period duration is divided by MEP size. However, Sale and Semmler (2005) and Fujiyama et al. (2012) did not find such a relationship. Thus, it is difficult to draw any conclusions about the interrelation of changes in behavioral parameters and changes of the duration of the silent period with age at this stage.

SICI is, unlike the silent period, mediated by GABA-A receptors and measured with paired pulse TMS. Studies found inconsistent results regarding the effect of age on SICI. Two studies report increased SICI with aging (Kossev et al., 2002; McGinley et al., 2010), while two other studies report decreased SICI (Marneweck et al., 2011; Heise et al., 2013), and the rest report no effect (Oliviero et al., 2006; Rogasch et al., 2009; Cirillo et al., 2010; Smith et al., 2011; Stevens-Lapsley et al., 2013). Also here, inconsistencies are probably caused by methodological differences such as the selection of TMS parameters and the muscles examined. Across studies there is a wide variety of methods to set the conditioning and test pulse stimulation intensities, complicating comparisons between studies. Moreover, there seems to be an interaction between the size of the upper extremity muscle and age on the amount of inhibition, with less SICI in old adults (Marneweck et al., 2011; Heise et al., 2013) or no age-related changes (Oliviero et al., 2006; Smith et al., 2011) when examining intrinsic hand muscles and greater SICI in old adults when examining the larger wrist flexors and extensors (Kossev et al., 2002; McGinley et al., 2010). Only one study (Heise et al., 2013) examined the correlation between the amount of SICI and behavioral measures. They reported that a weaker resting state SICI and less modulation in SICI during the movement preparation phase correlated with slower reaction times and alternating finger tapping.

Another type of inhibition is the inhibition of the corticospinal output to the antagonist muscle by afferent input from the agonist muscle (cortical reciprocal inhibition). This inhibition can be investigated by measuring the effect of peripheral afferent stimulation of the agonist muscle on TMS evoked MEP recorded in the antagonist muscle. At rest, the MEP in the extensor carpi radialis longus is inhibited by peripheral stimulation by about 40% in young adults, while no inhibition is apparent in old adults (Hortobágyi et al., 2006). This is interpreted as a decrease in cortical reciprocal inhibition with age, although influence of age-related changes in afferent input cannot be excluded. There are no studies linking cortical reciprocal inhibition with performance in motor tasks and it is also unclear if this form of inhibition is actually active during muscle contraction. It can be speculated, however, that the reduced cortical reciprocal inhibition has a role in the increased muscular coactivation seen in elderly subjects (Darling et al., 1989; Benjuya et al., 2004; Baudry et al., 2010).

The emerging picture is that aging causes a reorganization of cortical control of voluntary movement, with an increase in brain activation and decrease in cortical inhibition. However, most of the studies focused on healthy elderly aged between 60 and 80 years, and do not allow to clearly consider functional changes in the oldest old (>80 years) and the effect of co-morbidities on the proposed reorganization of cortical motor control. Moreover, it is not known whether the changes in inhibitory circuits are related to the increased brain activation. Similarly, it is not clear whether these changes in inhibitory circuits result from malfunction, or serve a compensatory purpose. To fully understand age-related changes in motor control, future studies should focus on the relationship between brain activation, the different forms of inhibition, and motor performance and determine if exercise modifies motor function and elements of the inhibitory system in a correlated manner. Combining TMS and brain imaging techniques would provide valuable data for the purpose of answering these questions.

## Relationship between structural and functional changes and postural control in aging

In the first section we argued that posture is not only controlled by spinal reflexes, but is also influenced by cortical structures. Hence, it seems inevitable that the age-related changes in the spinal and cortical systems, as reviewed in the previous sections, have an impact on postural control in old adults. Therefore, we will discuss the possibility that the structural and functional changes in the aging neuromotor system are linked to the deterioration of postural control in old adults (Figure 1), leading to the conclusion that aging causes a reorganization of postural control.

### Cortical structural changes and postural control

Many studies report that changes in cortical structures (brain atrophy, cortical thinning, and/or white matter hyperintensity) negatively correlate with performance in postural tasks. In these studies postural control was quantified based on single leg stance time (Baezner et al., 2008; Kido et al., 2010), postural sway (Sullivan et al., 2009; Kido et al., 2010; Van Impe et al., 2012), history of falls (Kerber et al., 1998; Ryberg et al., 2011), Tinetti gait and balance score (Kerber et al., 1998; Baloh et al., 2003), slowing of gait (Rosano et al., 2010; de Laat et al., 2012), and the short physical performance battery (SPPB) (Guttmann et al., 2000; Baezner et al., 2008; Ryberg et al., 2011).

Gray and white matter degeneration can interfere with postural control through the slowing in information processing speed. Rosano et al. (2012) demonstrated that the association between reduced prefrontal area volume and slowing of gait is explained by slower information processing, quantified by the Digit Symbol Substitution Test (DSST). Moreover, neuropsychological function mediates the relationship between white matter hyperintensities and choice stepping performance under dual task conditions (Zheng et al., 2012). In this study neuropsychological function was measured with the DSST, the trail making test, and the grooved pegboard test, together indicating information processing speed and attention capacity.

### Response time and postural control

Old adults respond to platform perturbations with longer muscle onset latencies that delay postural corrections (Nardone et al., 1995; Lin and Woollacott, 2002; Tokuno et al., 2010). Tilting or translating a horizontal platform on which people stand, elicit short (SLR) and long (LLR) latency responses. The age-related increase of 5 ms in SLR latencies is similar to the 3–7 ms difference in H-reflex latency (Sabbahi and Sedgwick, 1982; Nardone et al., 1995; Scaglioni et al., 2003). The prolongation in SLR latency can therefore be explained by reduced conduction velocity mediated by a loss in myelination (Verdu et al., 2000). Age-related delays in LLR are even more pronounced (20 ms), and thus cannot be explained by peripheral changes alone (Nardone et al., 1995; Allum et al., 2002). Consequently, there must be an increase in central processing time. Both the soleus SLR and LLR latencies are correlated with sway area after stance perturbation (Nardone et al., 1995), pointing to the behavioral significance of these response latencies and feedback control. Delayed postural corrections in old adults can thus be explained by functional deterioration in conduction and processing speed due to structural changes.

### Age-related reorganization of postural control

Whereas many studies have shown a greater activation of M1, prefrontal, and premotor areas and decreased cortical inhibition during upper extremity tasks, little is known about whether similar reorganizations occur during postural control. Zwergal et al. (2012) examined brain activation patterns with fMRI while young and old adults imagined that they were standing, walking and running. The age-related differences in brain activation were most prominent in standing, followed by walking and running. During imagined standing, a relative increase in activation with age was evident in numerous multisensory areas; the bilateral posterior insulae, superior and middle temporal gyri, inferior frontal gyri, fusiform and lingual gyri, MT/V5 areas, and the postcentral gyri. In young adults, activation of one sensory modality suppressed activation of other sensory modalities (Brandt et al., 1998; Laurienti et al., 2002). This phenomenon, called inhibitory reciprocal interaction of sensory systems, is thought to decrease with age (Townsend et al., 2006; Peiffer et al., 2009). A decrease in inhibitory reciprocal interaction could be used to explain the enhanced cortical sensory representation observed by Zwergal et al. (2012). It is suggested that this is a compensatory strategy for a decline in the unimodal sensory systems. In line with this hypothesis, Goble et al. (2012) observed 71% lower activation after muscle spindle stimulation in the right putamen of old compared to young adults, and the activity of this structure was positively related to performance on a proprioceptive joint position sense test in both age groups. The relationship between putamen activation and position sense was mediated by decreased white matter integrity in old adults.

In summary, similarly to manual tasks, old adults appear to use the increased activation strategy in postural tasks in compensation for structural and/or functional changes in other areas. Therefore, one element of the age-related reorganization of postural control is the increased brain activation. A clear distinction between the non-postural and postural data is that in postural tasks the age-related increase in activation occurred in sensory rather than motor areas involved in postural control, possibly due to the fact that the postural task was imaginary. A second element of the age-related reorganization of postural control is the decrease in inhibitory reciprocal interaction of sensory systems. However, whether motor cortical inhibition in its various forms (SICI, silent period, interhemispheric inhibition) also becomes weaker with aging during a postural task is not known and is a promising topic for future research.

One of the behavioral consequences of increased activation and decreased inhibition could be the use of a heightened coactivation strategy. Old adults execute many voluntary movements with increased antagonistic activity, including hand or arm movements (Seidler-Dobrin et al., 1998; Burnett et al., 2000; Klein et al., 2001), quiet standing (Nagai et al., 2011; Baudry and Duchateau, 2012), and when perturbed while standing (Manchester et al., 1989). The mechanisms underlying this coactivation are probably both spinal and cortical (for a review see: Hortobágyi and Devita, 2006). For example, potential mechanisms involve the reduction of spinal (Kido et al., 2004) and cortical (Hortobágyi et al., 2006) reciprocal inhibition with age, although these studies did not examine whether the increased antagonist activation was associated with either form of inhibition. Another potential cortical mechanism underlying the increased coactivation is the observed increased activation of motor and premotor brain areas in old adults. These areas include the antagonist representation area, resulting in greater activation of cortical neurons controlling the antagonistic muscle activity. As discussed before, this increased activation is probably the result of a shift in the balance between the activation of cortical inhibitory and excitatory circuits toward excitation in old adults. Although it seems plausible to link increased activation and decreased inhibition to coactivation, this relationship has not been confirmed.

Another behavioral consequence of the described functional changes could be the consistently higher interference of a cognitive task on postural control in old adults (Shumway-Cook et al., 1997; Marsh and Geel, 2000; Huxhold et al., 2006; Rapp et al., 2006). The cortical involvement during dual tasks in young adults has been investigated using fMRI and TMS techniques. For example, Wu et al. (2013) examined dual task-associated neural activity when subjects performed a finger tapping and a counting task. They observed that two cerebellar regions and the precuneus were only activated in dual task conditions but not during single task performance. This demonstrates that additional brain areas are recruited to integrate the two tasks. Others found no dual-task specific brain areas, but an increased activation of the areas that are active in both single tasks (Van Impe et al., 2011). TMS studies show that performing a motor task in upper (or lower) extremity increases corticospinal excitability (Hiraga et al., 2009) and decreases corticospinal inhibition (Sohn et al., 2005) to lower (or upper) extremity muscles. Although this activity-dependent coupling may mediate some of the dual task interference in dual motor tasks, it is not sensitive to several task modulations that do influence behavioral outcome measures (Hiraga et al., 2009). Therefore, it must be concluded that other factors are more important in dual-task interference. On the spinal level, a recent study suggests that increasing the difficulty of a cognitive task does not influence the H-reflex amplitude during a postural dual task in young and elderly adults (Baudry and Gaillard, 2013). Accordingly, the age-related differences in dual-task performance did not involve a change in the efficacy of homonymous Ia afferents to discharge motor neurons in young and elderly adults.

A common theory explaining dual task interference is the central capacity-sharing model. This model predicts that the resources are limited to execute both tasks concurrently (Tombu and Jolicoeur, 2003). Old adults might have a reduced residual capacity because of decreased availability of resources and increased neural recruitment (see sections Age-Related Functional Changes on the Cortical Level and Response Time and Postural Control). Van Impe et al. (2011) tested the hypothesis that the reduced residual capacity causes the age-related increase in dual task interference and found that both young and old adults were able to upregulate their brain activity in the common areas for dual- as compared to single-task performance. This means that, at least in these tasks, central capacity limited young and old adults to a similar extent. However, the cognitive task (arithmetic addition) might not have been challenging enough to reach the central capacity limit since there were no age-related differences in dual task costs, nor in brain activity associated with the cognitive task.

Van Impe et al. (2012) examined the neural correlates of dual task performance involving a postural task in young and old adults. The increase in the error rate while performing a mental rotation task in standing as compared to sitting was higher in old compared with young adults. Interestingly, in old adults activation in the left lingual gyrus during the mental rotation task while lying in the scanner correlated with the change in performance from the seated to the standing position outside of the scanner (*r* = −0.83, *p* < 0.05), so that more activation was associated with better performance. This finding is in contradiction with the hypothesis that increased neural recruitment leads to reduced residual capacity and results in greater dual task interference, but is in line with the theory that the increased activation seen in old adults acts as a compensatory mechanism.

In summary, the age-related reorganization in cortical motor control during voluntary tasks, characterized by greater brain activation and reduced inhibition, is also present during postural tasks. Further research will determine whether in addition to sensory areas, age also affects the function of motor areas during postural tasks. Further, there is the need to better understand whether the age-related increase in antagonist muscle coactivation during postural tasks exacts functional costs. Although theories have been proposed that greater activation mediates the age-related increase in dual task interference, so far scientific evidence is lacking.

### Spinal vs. supraspinal postural control mechanisms

As discussed in section Cortical Structural Changes and Postural Control, old compared to young adults appear to have a different relative contribution of supraspinal and spinal inputs to the gain control of motor output during a voluntary task. Also during postural tasks, the control of spinal reflexes differs between young and old adults. In young adults, greater postural instability is associated with greater down regulation of the H-reflex (Koceja and Mynark, 2000), presumably through presynaptic inhibition (Katz et al., 1988). It is thought that the downregulation in the H-reflex prevents postural disturbance (Llewellyn et al., 1990). In old adults, H-reflexes are lower and the modulation of the H-reflex with increasing postural task difficulty is either the opposite of what is observed in young adults (upregulation) (Koceja et al., 1995) or the reflex modulation is absent (Koceja and Mynark, 2000). According to Koceja and Mynark (2000) the reflex upregulation or the absence of it is accompanied by a decreased ability of old adults to modulate presynaptic inhibition. In contrast, Baudry and Duchateau (2012) observed greater upregulation of presynaptic inhibition in old adults compared to young adults with increasing task difficulty, suggesting a feedforward mechanism that reduces spinal contribution and thus increases supraspinal contribution in difficult postural tasks. Furthermore, the amount of presynaptic inhibition was associated with sway amplitude in the sagittal plane and coactivation of leg muscles in old adults, so that greater Ia presynaptic inhibition was accompanied by greater sway amplitude and greater coactivation, suggesting an age-related depression of the inputs from muscle afferents compensated by an augmentation of coactivation among leg muscles during the control of upright stance (Baudry and Duchateau, 2012). This could be related to the greater level of muscle activation that limits muscle lengthening and shortening and therefore the relevance of spindle afferents (Baudry et al., 2012). Furthermore, a recent study reports lower efficacy of Ia afferents to discharge spinal motor neurons accompanied by greater corticospinal excitability in elderly adults, indicating an increased contribution of the descending drive in controlling soleus activity during upright standing with aging (Baudry et al., 2014). In conclusion, studies suggest that an additional element of age-related reorganization of postural control is an alteration in the control of presynaptic inhibition in postural tasks, although the direction of this alteration is inconsistent and/or the modulating extrinsic (e.g., postural task, task difficulty) and intrinsic factors (e.g., back pain, impaired vision) are not yet known. Furthermore, to date, only cross-sectional studies are available so that there are no data showing changes of neural control mechanisms with age.

Another approach to examine the role of spinal reflexes in the control of standing posture is to move the support surface on which subjects stand. Most studies showed that the amplitude of the early response was smaller and the late response was higher in old compared with young adults (Allum et al., 2002; Lin and Woollacott, 2002). The observation of smaller early responses is consistent with the age-related decrease in H-reflex amplitude in the soleus (Koceja et al., 1995; Baudry and Duchateau, 2012). However, smaller H-reflexes were not observed in the tibialis muscle at rest or during small voluntary contractions (Klass et al., 2011). Therefore, the reduced postural response in the tibialis anterior may indicate a specific downmodulation in upright standing. Furthermore, as the early responses (short- and medium latency responses) are mediated via spinal reflex circuits but long-latency responses also involve supraspinal structures (Taube et al., 2006), the shift toward later responses with aging may suggest a change in the contribution of spinal and supraspinal mechanisms when standing posture is experimentally perturbed.

Together with the findings of increased cortical activation during postural tasks and the increased dual task interference with aging, there is some evidence suggesting that old compared with young adults rely less on spinal reflexes and more on cortical activation than young adults for postural control, although this remains to be fully explored. The reason for a reduced reliance on spinal reflexes is not known. Among the candidates is the prominent reduction in conduction velocity and degeneration of somatosensory receptors (Shaffer and Harrison, 2007; Goble et al., 2009) that contribute to a decreased relevance of afferent inputs and therefore reduces the reflex efficiency of spinal reflex in postural control. Although these impairments also affect supraspinal responses, we speculate that the high degrees of freedom in the supraspinal mechanism afford old adults flexibility to compensate for neuromuscular impairments. Another possible explanation for a reduced reliance on spinal reflexes is the difference in relative task difficulty; the same postural task is more difficult for old than for young adults. Indeed, when young individuals encounter increasingly difficult postural tasks, spinal reflexes become attenuated (Koceja and Mynark, 2000).

## Plasticity of postural control mechanisms

Balance training improves old adults' postural control (Jacobson et al., 2011; Halvarsson et al., 2013). However, neural correlates of balance training, demonstrating plasticity of postural control mechanisms, have mostly been studied in young subjects. Several electrophysiological studies have found task specific decreased cortical excitability induced by balance training, indicating cortical plasticity (Beck et al., 2007; Taube et al., 2007; Schubert et al., 2008). In one study, the training induced changes in cortical measures were correlated with the changes in postural performance so that subjects who had a greater reduction in cortical excitability, improved their postural performance more (Taube et al., 2007). This study also showed a phase-specific adaptation of the soleus H-reflex. Balance training decreased the amplitude of the H-reflex elicited at the time of the LLR but not at the SLR. The authors favored the explanation that supraspinally induced presynaptic inhibition was selectively increased at the time of the LLR that was previously shown to be transcortically mediated (Taube et al., 2006). Thus, adaptation of supraspinal processing in response to balance training seems to be responsible for both alterations of spinal reflex circuits and descending motor commands.

Imaging studies support the idea that cortical and subcortical adaptations play a putative role in improving postural control after balance training. In a cross-sectional study, the hippocampus structure was different between female dancers and slackliners and recreationally active females (Hufner et al., 2011). This may indicate that long-term training involving demanding postural tasks leads to structural changes of the brain. Recent imaging data obtained during balance training support this view and highlight that structural changes in gray and white matter volume in response to balance exercises may occur very rapidly (Taubert et al., 2010). The authors recorded brain images after every second training session and displayed rapid transient gray matter changes in sensorimotor areas (after 2 training sessions) and more slowly evolving increases in parts of the orbitofrontal cortex (after 6 training sessions). Interestingly, there was a positive linear correlation between gray matter expansion in the left supplementary motor area (SMA) and the ability to balance on the training device, an unstable moving platform. In a subsequent study involving the same subjects and the same structural data, the authors demonstrated an association between these structural gray matter alterations and changes in functional connectivity of prefrontal and supplementary-motor areas (Taubert et al., 2011). Therefore, there is strong evidence that the morphological adaptations are functionally relevant with respect to alterations in postural control. Furthermore, these studies emphasize that structural adaptations occur very rapidly in response to balance training. Finally, one single balance training session of 45 min, incorporating 15, 30-s-long balancing trials resulting in 7.5 min effective training time, is sufficient to induce macroscopic structural changes in areas belonging to the vestibular cortical system (Taubert et al., 2013 OHMB). Thus, both electrophysiological and imaging studies illustrate that cortical adaptations occur in response to balance training and that these changes are of high functional relevance.

To the best of our knowledge, there are no studies that examined age-related differences in cortical adaptations to balance interventions. There are, however, two recent papers that provide preliminary insights into the neural plasticity associated with balance training in old adults (Burciu et al., 2013; Sehm et al., 2014). In these papers, the healthy old adults served as controls for Parkinson and cerebellar patients. Sehm et al. (2014) report a positive linear correlation between changes in left hippocampus volume and balance performance after training of a dynamic balancing task. Burciu et al. (2013) report a trend for gray matter volume increases in several cortical (occipital cortex and superior temporal gyrus) and subcortical (left putamen and hippocampus, right cerebellum, VIIIb) structures, after 2 weeks of balance training consisting of weight shifting exercises. Although a direct comparison with young adults is missing, these results do suggest that structural plasticity is still present in the aging brain.

Considering spinal adaptations, the limited data suggest that the nature of adaptation to exercise training is similar in young and old adults. For instance, Mynark and Koceja (2002) tested subjects' ability to down train the soleus H-reflex because high H-reflex amplitude tends to destabilize upright stance. Old compared with young adults were able to reduce the H-reflex to a similar extent. At the same time, postural stability improved in both groups. In another study with elderly participants, Granacher et al. (2006) demonstrated changes in postural (spinal) reflex responses that were accompanied by an improved ability to compensate for postural disturbances. Thus, the central nervous system of elderly people shows plasticity in response to postural exercises. It is not yet known whether such adaptations differ in magnitude or follow a different time course compared to in young adults.

## Conclusions

In young adults it is established that the cerebral cortex contributes to postural control of undisturbed and disturbed standing. Therefore, the degeneration and accompanying functional changes in the brain seen in motor control of simple manual tasks are also operational under postural tasks and likely to influence postural control. Indeed, gray and white matter loss with aging is associated with decreased performance in postural tasks. Moreover, there is a reorganization of cortical and spinal control of posture with aging including increased cortical activation, cortical disinhibition, and differential control of spinal reflexes. The structure-function-behavior model in Figure 1 highlights the need for future studies to incorporate behavioral measures to document how these age-related structural and functional changes influence postural control. In general, we recommend future studies to include at least two of the three domains in the model, to be able to examine associations between domains. This method can for example be applied to test the hypotheses that the age-related spinal and cortical functional changes underlie the greater amount of co-activation and decreased dual task performance seen in old adults during postural tasks. Eventually, the plasticity of the aging neuromotor system controlling posture should be examined using balance training.

### Conflict of interest statement

The authors declare that the research was conducted in the absence of any commercial or financial relationships that could be construed as a potential conflict of interest.

## Appendix I

### Abbreviations

BOLD: blood-oxygen-level dependence
DSST: Digit Symbol Substitution Test
EEG: electroencephalography
EMG: electromyography
fMRI: functional magnetic resonance imaging
H-reflex: Hoffmann's reflex
LLR: long latency reflex
M1: primary motor cortex
MEP: motor evoked potential
MLR: medium latency reflex
MRI: magnetic resonance imaging
S1: primary sensory cortex
SICI: short-interval intracortical inhibition
SLR: short latency reflex
SMA: supplementary motor area
SPPB: short physical performance battery
TMS: transcranial magnetic stimulation

### Search terms

postural control, balance, aging, older adults, cortical, fMRI, EEG, and TMS.
